# Serum alpha-CGRP levels are increased in COVID-19 patients with headache indicating an activation of the trigeminal system

**DOI:** 10.1186/s12883-023-03156-z

**Published:** 2023-03-17

**Authors:** Gabriel Gárate, María Toriello, Vicente González-Quintanilla, Sara Pérez-Pereda, Jorge Madera, Marta Pascual, José Manuel Olmos, Julio Pascual

**Affiliations:** 1 Service of Neurology, University Hospital Marqués de Valdecilla, Universidad de Cantabria and IDIVAL, Av. Valdecilla s/n, Santander, Cantabria, 39008 Spain; 2 Service of Gastroenterology, University Hospital Marqués de Valdecilla, Universidad de Cantabria and IDIVAL, Santander, Spain; 3 Service of Internal Medicine, University Hospital Marqués de Valdecilla, Universidad de Cantabria and IDIVAL, Santander, Spain

**Keywords:** COVID-19, CGRP, alpha-CGRP, Headache trigeminal system

## Abstract

**Background:**

Headache is among the most frequent symptoms of acute COVID-19 infection. Its mechanisms remain obscure, but due to its migraine-like characteristics, the activation of the trigeminal system could account for its underlying pathophysiology.

**Methods:**

Our aim was to compare the serum levels of CGRP, as a theoretical marker of trigemino-vascular activation, in 25 COVID-19 inpatients with lung involvement experiencing headache, against 15 COVID-19 inpatients without headache and with those of 25 matched healthy controls with no headache history.

**Results:**

Morning serum alpha-CGRP levels, as measured by ELISA (Abbexa, UK), were increased in COVID-19 patients with headache (55.2±34.3 pg/mL) vs. controls (33.9±14.0 pg/mL) (p < 0.01). Alpha-CGRP levels in COVID-19 patients without headache were also significantly increased (43.3 ± 12.8 pg/mL; p = 0.05) versus healthy controls, but were numerically lower (-28.2%; p = 0.36) as compared to COVID-19 patients with headache.

**Conclusion:**

CGRP levels are increased in COVID-19 patients experiencing headache in the acute phase of this disease, which could explain why headache frequently occurs in COVID-19 and strongly supports a role for trigeminal activation in the pathophysiology of headache in this viral infection.

## Introduction

Headache is one of the most frequent initial symptoms in COVID-19 infection [1–5]. Even though headache tends to be bilateral it usually shows migraine properties, such as moderate-severe intensity or a pulsating quality. A recent study has demonstrated that in approximately a fifth of patients who present headache during the acute phase of COVID-19, it becomes persistent. Moreover, in many of these cases headache remains with a migraine-like phenotype and the persistence of headache after COVID-19 infection is higher in patients with a previous history of migraine [6]. Although the pathophysiology of COVID-19 headache is yet unclear, it is tempting to propose that the inflammatory response associated with COVID-19 infection could activate the trigeminal system, which would account for the COVID-19 migraine-like headache. It is true that there is not a fully reliable marker for the activation of the trigeminal system, but a number of studies (see reference 7 for a recent review) have shown that CGRP is increased in acute migraine attacks [8, 9] and interictally in chronic migraine (10,11) in several human compartments, including serum [7–11], saliva [12] and cerebrospinal fluid [13]. The objective of this study was to compare the serum levels of CGRP, as a theoretical marker of trigemino-vascular activation, in COVID-19 inpatients experiencing headache versus COVID-19 patients and healthy controls with no headache history.

## Methods

Alpha-CGRP levels were assessed from early morning blood samples in COVID-19 inpatients in their acute phase experiencing headache vs. COVID-19 inpatients without headache and matched healthy controls. Healthy controls were doctor and nurses from our hospital and family members of COVID-19 patients. Healthy controls were included if they had no history of migraine and of other active diseases and if they took no medication. To avoid storage influence, samples of COVID-19 and healthy controls were taken at the same time. All methods were carried out in accordance with relevant guidelines and regulations and declaration of Helsinki. The study was approved by our institutional Ethics Committee and written informed consent was obtained from all participants. We excluded COVID-19 patients with severe comorbidities, chronic pain conditions, with cognitive impairment or those admitted in the Intensive Care Unit. Samples, both from controls and patients, were obtained from January to September 2021. The blood was collected from the antecubital vein, allowed to clot, afterwards serum was separated after centrifugation for 10 min at 3500 rpm. Aliquots were immediately stored at -80ºC until assayed. Alpha-CGRP levels were determined using a commercial ELISA (Abbexa, UK) strictly following manufacturer’s instructions. The detection limit of the assay was 3.12pg/mL. CGRP content was described as mean ± SDs. Comparisons were established using the non-parametric Mann-Whitney test as CGRP measurements were not normally distributed.

## Results

Alpha-CGRP levels were assessed from 25 healthy controls (mean age = 56.0±16.5 years; range 27–85 years; 53.3% females), 25 COVID-19 inpatients experiencing headache (mean age = 55.8±15.7 years) and 15 COVID-19 inpatients without headache (mean age 57.8 ± 13.7; range = 36–86; 53.3% females). All patients had active lung involvement directly related to COVID-19 and had headache at the time samples were collected. Headache was at least moderate and required symptomatic treatment. Headache was bilateral in 21 cases and was accompanied by gastrointestinal manifestations in ten cases. There was just one patient with a previous history of primary headache (migraine). Patients without headache did not experience headache during their whole hospital stay. In this group there was also one patient with a history of migraine Main comorbidities of COVID-19 patients are summarized in Table 1. Within the headache group fifteen (60%) patients were receiving methylprednisolone (20–80 mg/24 h) and all 25 were being treated with analgesics (at least 500 mg paracetamol every 8 h). Regarding the non-headache COVID-19 subjects, ten (66%) patients were receiving methylprednisolone (20–80 mg/24 h) and 3 had been treated with paracetamol due to fever or pain in other areas of the body the day before blood samples were taken.

**Table 1:** Main comorbidities in COVID-19 patients

Disease	With headache n (%)	Without headache n (%)
Arterial hypertension	12 (48%)	5 (33%)
Obesity	11 (44%)	5 (33%)
Hypercholesterolemia	6 (24%)	5 (33%)
Diabetes	5 (20%)	2 (13%)
Hypothyroidism	2 (8%)	2 (13%)

**Fig. 1:**
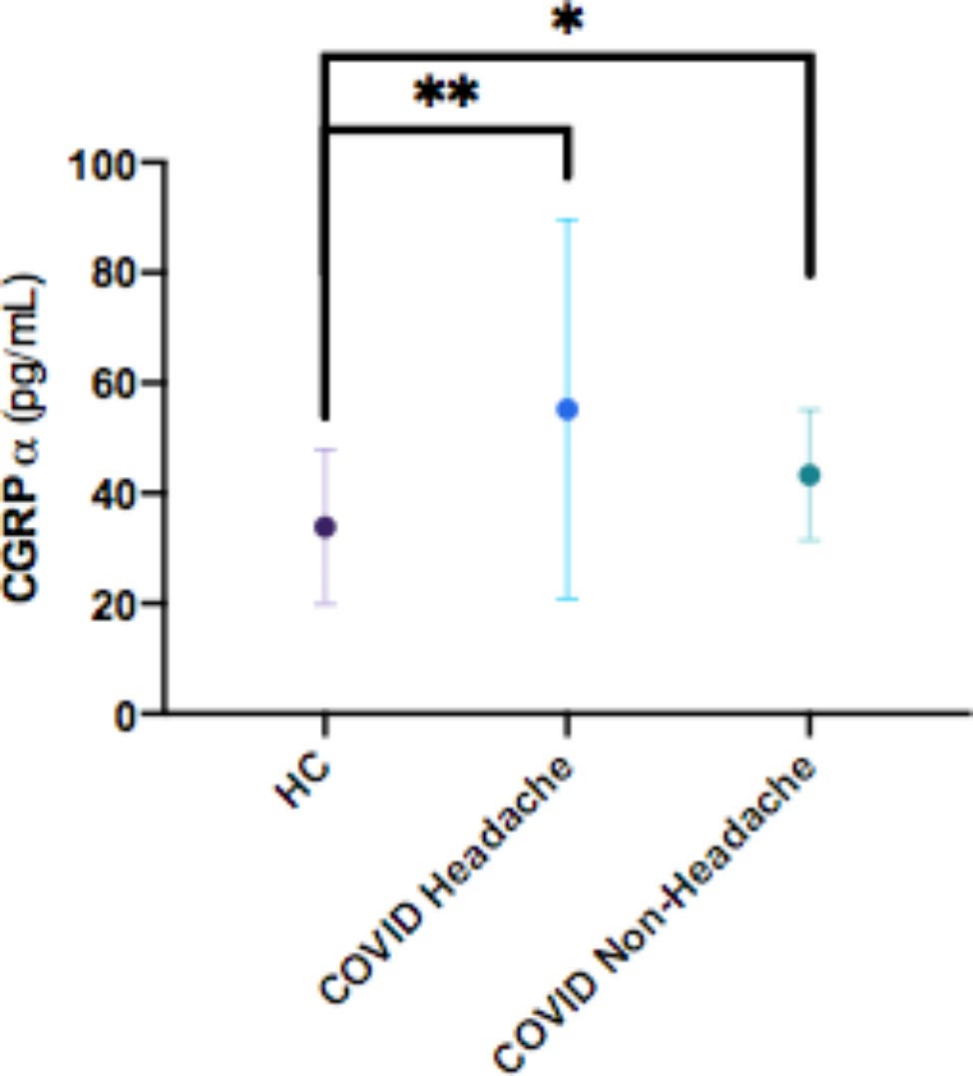
Comparison of alpha-CGRP levels between healthy controls and COVID-19 inpatients with and without headache. Data are presented as mean±standard deviation **p < 0.01; *p = 0.05

Alpha-CGRP levels were significantly elevated in COVID-19 patients with headache (55.2±34.3 pg/mL) vs. controls (33.9±14.0 pg/mL) (+ 38,5%; p < 0.01) (Fig. 1). Alpha-CGRP levels were also significantly increased (p = 0.05) in COVID-19 patients without headache versus healthy controls (Fig. 1). There was no difference in alpha-CGRP levels of the 25 patients without (51.2 ± 31.4 pg/mL) versus the 15 patients with methylprednisolone (50.5 ± 27.3 pg/mL).

## Discussion

The main finding of this study is that alpha-CGRP levels are significantly increased in COVID-19 inpatients with acute headache as compared to healthy controls. Serum alpha-CGRP levels were almost 30% higher in COVID-19 inpatients with headache as compared to a similar group of COVID-19 patients without headache. These data indicate, as recently proposed for the COVID-10 anosmia [14], that trigemino-vascular activation is involved in the pathophysiology of acute headache in COVID-19 patients and, as happens in chronic migraine [10, 11], invite to suggest a role of a rather permanent activation of the trigeminal system in the pathophysiology of persistent post-COVID-19 headache.

To our knowledge, serum CGRP levels have been measured in COVID-19 patients in three previous works with heterogeneous results [15–17,]. In the study by Ochoa-Callejero et al., CGRP levels were decreased in COVID-19 patients versus healthy controls, but the samples of healthy volunteers were obtained “before the initiation of the pandemia” and were not matched for age. Just as an example, the mean age of controls in that study was 41 years and that of symptomatic inpatients (those who would be theoretically comparable to our patients) was 81 years [15]. Additionally, they do not report whether their COVID-19 subjects had headache and their serum CGRP levels of control subjects was unusually high (an average of 220.7 pg/mL, when most published studies show CGRP levels in healthy controls below 50 pg/mL) [7, 10, 11]. In a second study, Bolay et al. determined serum CGRP in COVID-19 inpatients with and without headache. The levels of CGRP were similar in both groups, but they did not analyze, for instance, the influence on CGRP levels of acute analgesics or steroids and, what is most important, there was no control group of healthy subjects [16]. Our group has very recently found an increase in both alpha- and beta-CGRP levels in serum of COVID-19 inpatients as compared to healthy controls [17].

Even considering that we obtained data from a limited number of subjects, the increase in alpha-CGRP found here was clear even in the presence of drugs such as corticosteroids or analgesics, which could theoretically diminish CGRP release, and supports a role for this neuropeptide in the headache manifestations of COVID-19 and, together with the recently described specific increase in beta-CGRP serum levels in COVID-19 patients with diarrhea (18), suggests that CGRP could be one of the molecules released in the cytokine storm induced by the COVID-19 infection. CGRP is known to enhance interleukin-6 production, the main biomarker of COVID-19 severity, which has even brought the proposal that CGRP antagonists could be of help in COVID-19 infection [19, 20]. Even though the significant increase in alpha-CGRP serum levels found here fits well with the proposal of an acute activation of the trigemino-vascular system explaining migraine-like headache seen in COVID-19 patients [14], we cannot rule out that the release of CGRP could occur at distance, for instance in the damaged nerves of the disease lungs of our patients [17]. As happens for increased beta-CGRP, which correlates with the presence of diarrhea [18], an excess of alpha-CGRP released from the lung or other body organs could also induce headache at distance by stimulating nerve terminals in the trigemino-vascular system.

## Conclusion

Alpha-CGRP levels are increased when compared to healthy subjects with no headache history in COVID-19 inpatients admitted due to lung involvement and mainly in those experiencing headache in the acute phase of this disease, which could explain why headache (and other symptoms, such as anosmia) frequently occur in COVID-19 and supports a role for trigeminal activation in the pathophysiology of headache in this viral infection.

## Data Availability

The datasets used and/or analysed during the current study are available from the corresponding author on reasonable request.

## Abbreviations

CGRP: calcitonin gene-related peptide
COVID-19: coronavirus disease 2019
